# Variations in local calcium signaling in adjacent cardiac myocytes of the intact mouse heart detected with two-dimensional confocal microscopy

**DOI:** 10.3389/fphys.2014.00517

**Published:** 2015-01-12

**Authors:** Karin P. Hammer, Felix Hohendanner, Lothar A. Blatter, Burkert M. Pieske, Frank R. Heinzel

**Affiliations:** ^1^Department of Cardiology, Medical University of GrazGraz, Austria; ^2^Department of Internal Medicine II, University Hospital RegensburgRegensburg, Germany; ^3^Molecular Biophysics and Physiology, Rush Medical College, Rush UniversityChicago, IL, USA; ^4^Department of Cardiology, Charité-Universitaetsmedizin BerlinBerlin, Germany

**Keywords:** whole heart, calcium cycling, excitation contraction coupling, 2D confocal microscopy, Langendorff perfused heart, cardiac myocytes, local dyssynchrony, intercellular communication

## Abstract

Dyssynchronous local Ca release within individual cardiac myocytes has been linked to cellular contractile dysfunction. Differences in Ca kinetics in adjacent cells may also provide a substrate for inefficient contraction and arrhythmias. In a new approach we quantify variation in local Ca transients between adjacent myocytes in the whole heart. Langendorff-perfused mouse hearts were loaded with Fluo-8 AM to detect Ca and Di-4-ANEPPS to visualize cell membranes. A spinning disc confocal microscope with a fast camera allowed us to record Ca signals within an area of 465 μm by 315 μm with an acquisition speed of 55 fps. Images from multiple transients recorded at steady state were registered to their time point in the cardiac cycle to restore averaged local Ca transients with a higher temporal resolution. Local Ca transients within and between adjacent myocytes were compared with regard to amplitude, time to peak and decay at steady state stimulation (250 ms cycle length). Image registration from multiple sequential Ca transients allowed reconstruction of high temporal resolution (2.4 ± 1.3 ms) local CaT in 2D image sets (*N* = 4 hearts, *n* = 8 regions). During steady state stimulation, spatial Ca gradients were homogeneous within cells in both directions and independent of distance between measured points. Variation in CaT amplitudes was similar across the short and the long side of neighboring cells. Variations in TAU and TTP were similar in both directions. Isoproterenol enhanced the CaT but not the overall pattern of spatial heterogeneities. Here we detected and analyzed local Ca signals in intact mouse hearts with high temporal and spatial resolution, taking into account 2D arrangement of the cells. We observed significant differences in the variation of CaT amplitude along the long and short axis of cardiac myocytes. Variations of Ca signals between neighboring cells may contribute to the substrate of cardiac remodeling.

## Introduction

Synchronous calcium (Ca) cycling is a fundamental part of excitation contraction coupling (ECC) in cardiac myocytes. Dyssynchronous Ca release within a single myocyte has been observed in various diseased conditions in animal models as well as in human cardiac myocytes from diseased patients and has been proposed as a possible mechanism of cellular contractile dysfunction (Louch et al., 2004; Song et al., 2006; Heinzel et al., 2008). While the spatial distribution of Ca release has been studied extensively (see Heinzel et al., 2011 for review), variation in local Ca removal within individual cardiac myocytes has only recently been systematically investigated. We have described increased dyssynchrony of intracellular cytosolic Ca removal which was linked to non-uniform sarcomere relengthening and cardiac malfunction in different models of heart failure and in human cardiac myocytes (Hohendanner et al., 2013).

Observations in isolated cardiac myocytes cannot always be readily extrapolated to the whole organ where myocytes are functionally connected with each other forming an active syncytium (Joyner et al., 1983; Rohr et al., 1997; Spach and Boineau, 1997). However, also in the intact heart dyssynchronous Ca handling between larger areas of the heart can cause arrhythmogenic events (Myles et al., 2012) that in turn can lead to mechanical dysfunction of the heart. Furthermore, on a larger scale, mechanical dyssynchrony between different regions of the regularly beating heart—e.g., caused by bundle branch block—is a well-known promoter of HF and has led to cardiac resynchronization therapy as an established HF therapy (Yu et al., 2006; Stevenson et al., 2012).

The role of intercellular communication has been the center of interest during arrhythmogenic conditions (Wasserstrom et al., 2010; Li et al., 2012; Myles et al., 2012) In healthy intact myocardial tissue neighboring cells influence each other and provide a safety margin of electrical stability in case of spontaneous Ca release (SCR) or irregular electrical activity in individual cells (Houser, 2000; Bers, 2008; Xie et al., 2010; Plummer et al., 2011). At the tissue level Ca transients in cardiac myocytes have also been largely studied in arrhythmogenic conditions such as SCR (Aistrup et al., 2011; Shiferaw et al., 2012) or cardiac alternans (Aistrup et al., 2006; Hammer et al., 2014b; Wang et al., 2014). In the regular beating healthy heart the rapid spread of electrical activation ensures a near simultaneous initiation of Ca release within neighboring cardiac myocytes (Rubart et al., 2003). However, the shape of the resulting Ca transient depends on a variety of factors including the distribution of the intracellular T-tubule system and the activity of sarcolemmal and sarcoplasmic Ca carriers within the cardiac myocytes (Bers, 2008; Heinzel et al., 2011). The complexity of local Ca regulation within individual cells may give rise to variability in Ca kinetics between adjacent cardiac myocytes that may impact on the function of the heart as a whole. However, little is known about the synchrony of Ca signals between neighboring cardiac myocytes in the intact regularly beating heart. Furthermore, it is controversial in which way gap junctions actively contribute to Ca signaling between connected cells (Li et al., 2012). As gap junctions preferentially form at the transversal side between cardiac myocytes, variability of Ca transients between neighboring cells may depend on their orientation toward each other.

Confocal line scan imaging is used to record spatially resolved local Ca signals in isolated cells and has also been applied to cardiac myocytes in the epicardial layer of intact hearts (Prentice et al., 1990; Escobar et al., 2012). Given the physiological anisotropy of the heart, 2D imaging has the advantage over line scan imaging that it provides simultaneous information on neighboring cardiac myocytes along the longitudinal and also the transversal axis of an index cell. However, limited by the scanning speed and signal-to-noise ratio of current imaging systems, the temporal resolution of confocal 2D imaging is in the order of one magnitude lower than for line scans, which may limit the sensitivity for local Ca dyssynchrony in the heart.

In the present study, we therefore investigated local differences in Ca signaling in the intact heart using line scan imaging as well as a novel approach of fast 2D imaging. This method allowed us to differentiate local Ca gradients and kinetics in relation to the geometric orientation of adjacent cells. Our results revealed variation in the kinetics of Ca transients between adjacent cardiac myocytes as well as local differences in Ca regulation in longitudinal and transversal direction within the cardiac myocytes.

## Methods

### Animal models

Animal housing and breeding as well as all experimental procedures involving animals were approved by the local animal care and use committees according to the *Guide for the Care and Use of Laboratory Animals* prepared by the US National Academy of Sciences (National Institutes of Health publication No. 85-23, revised 1996). All experiments were performed on adult male C57BL/6 mice.

### Line scan recording and analysis

Adult male C57BL mice were sedated using isoflurane and sacrificed by cervical dislocation. The hearts were quickly removed and placed on the stage of a Nikon A1R (Nikon Corporation, Melville, NY, USA) while perfused via a Langendorff apparatus. Hearts were perfused with normal Tyrode's solution (NT, containing: NaCl 135 mM, KCl 5.4 mM, CaCl_2_ 2 mM, MgCl_2_ 1 mM, Glucose 10 mM, and HEPES 10 mM, pH 7.35) for 5 min at room temperature (RT). Subsequently Rhod-2 AM (20 μM) was added to the solution, recirculated for 40 min and washed with NT for 10 min. Rhod-2 was excited at 543 nm and emission was collected at wavelengths >600 nm. Action potentials (APs) were elicited due to spontaneous depolarization of the heart (~1.4 Hz) or by electrical stimulation at 3 Hz via epicardial electrodes. The fluorescence signal was collected at 512 lines s^−1^ using a ×60 oil-immersion objective lens (NA = 1.49) with a pixel size between 0.17 and 0.39 μm. The scan line was placed along the transversal axis of 4–8 adjacent myocytes. Individual cells were identified due to their separation by regions that showed only little change of fluorescence during Ca transients.

Line scan data from consecutive Ca transients was averaged and fluorescence traces are presented as F/F_0_ where F_0_ is the initial resting fluorescence. Local Ca transients and Ca decay constants along the scan line were quantified as previously described (Hohendanner et al., 2013).

### Heart preparation and langendorff perfusion for 2D imaging

The procedure of heart preparation for the 2D Nipkow setup was adapted from a previously described protocol (Hammer et al., 2014a). Adult male C57BL mice were sedated using isoflurane and sacrificed by cervical dislocation. Hearts were quickly excised, cannulated, and connected to a Langendorff perfusion system. The hearts were initially perfused at 37°C with NT until a regular heartbeat was reached. To slow the intrinsic heart rate the SA-node was removed and the AV-node crushed.

Loading of the hearts with the fluorescent dyes Fluo8-AM (MoBiTec GmbH, Göttingen, Germany; 3.75 μM; 30 min) for detection of Ca transients (CaT) and Di-4-ANEPPS (VWR International, Vienna, Austria; 1 μM; 7 min) for the visualization of plasma membranes as well as all subsequent recordings were performed at RT to reduce dye leakage and increase stability of the preparation throughout the course of an experiment. Perfusion pressure was kept constant at 70–90 mmHg by adjusting the flow rate accordingly. To exclude motion artifacts during the recording of CaT, contraction was inhibited by addition of blebbistatin (Biomol GmbH, Hamburg, Germany) with a concentration of at least 10 μM, which was increased up to 20 μM if residual movements were detected.

### Confocal 2D microscopy

The heart was fixed to a custom-built perfusion chamber on a microscope (Nikon Eclipse TE-2000U) equipped with a Nipkow spinning disc confocal scan unit (Visitech International, Yokogawa CSU-10). The microscope stage accommodated a glass bottom petri dish as organ bath and the holders for the cannula, stimulator, EKG pellet-electrodes, and aspirator. The heart was placed in the chamber with a slight angle to assure that it touched the glass bottom in the center region of the petri dish, where the epicardial layer of the left or right ventricle could be measured with no further fixation needed. The aspirated perfusion solution was either discarded or recirculated, e.g., during dye loading. Electrical stimulation was delivered via two silver electrodes placed on the base of the heart by applying square pulses with a frequency of 4 Hz/basic cycle length 250 ms (BCL). The applied stimulation frequency has to be faster than the intrinsic residual frequency of the conduction system in the heart to ensure a controlled and stable BCL during the experiment. Here we chose the lowest possible frequency that would not lead to irregularities in Ca cycling with normal perfusion solution and with isoproterenol (ISO) (Hammer et al., 2014b), which was 4 Hz in our experimental setup. Fluorescent signals were recorded using an Ar-laser for excitation at 488 nm. For detection of the emitted signal at 515 nm, a fast and highly sensitive 16 bit, 5.5-megapixel camera (pco.edge sCMOS; Visitron Systems; Germany) was used. CaT from the whole heart were recorded with a frame rate of 55 frames per second and a frame size of 465 μm by 315 μm, which covered about 12–20 adjacent cells. Simultaneously, the emitted signal from the Di-4-ANEPPS was recorded to be able to discern single cells and recognize cell borders.

A far field electrical, ECG-like signal was recorded continuously via two epicardial electrodes to monitor the intrinsic heart rate and potential rhythm irregularities. After fixing the heart on the microscope stage, it was perfused until a stable spontaneous heart rate was reached reflecting functional integrity of the myocardium. After dye loading and inhibiting contractions with blebbistatin, the hearts were stimulated at a frequency of 4 Hz for at least 1 min before a recording was started to ensure that steady state was reached.

To record β-adrenergic effect isoproterenol (ISO, Sigma-Aldrich Handels GmbH, Vienna, Austria; 10 μM) the hearts were perfused with NT containing ISO and measurements started after a noticeable increase in intrinsic frequency. The baseline frequency in NT solution was usually at about 2 Hz and increased to 3–3.5 Hz immediately after ISO reached the heart via the perfusion system. After another 5 min of ISO perfusion the same stimulation protocol as described above was applied. With the aim to record background fluorescence, we measured extracellular myocardial space adjacent to the recorded cardiac myocytes. This approach has proven difficult, as a Nipkow disk imaging is known to forfeit confocality when used on thick specimen. In this case, the fluorescence from out-of-focus cells might pass through neighboring pinholes and create cross talk between adjacent pinholes. This loss of confocality leads to a considerable overestimation of background signal. In a second approach, we quantified background fluorescence from unstained hearts before loading. For this approach we have positioned the hearts on the microscope as usual and tried to record from the epicardial surface before loading with the dyes. However with this approach, we were not able to detect fluorescence from the hearts.

### Data acquisition and signal extraction

Ca signals were recorded from the epicardial layer of cardiac myocytes of the central region of the left ventricle. An area with clearly visible cells was positioned in the field of view and steady state transients recorded for 9 s (500 frames). The intensity profiles of neighboring cells were acquired by placing regions of interest (ROIs) and extracting the values for further analysis. The ROIs were always positioned following a predefined scheme depicted in **Figure 3B**. In detail, an index cell was chosen and the intensity of the whole cell taken (Z0_g). Next, square shaped ROIs with a size of 2.56 × 2.56 μm (8 × 8 pixels) were positioned in the center of the index cell, the center of the cell bordering the index cell at the short side and the center of the cell bordering at the long side. Furthermore, ROIs were placed at the subsarcolemmal region of each of the cells. The ROIs within a single cell (INTRA) had a distance of 45 ± 6 μm from center to the subsarcolemmal ROI at the transversal axis and 80 ± 6 μm from center to the subsarcolemmal ROI at the longitudinal axis of the cell. The subsarcolemmal ROIs of neighboring cells were in close proximity (9.4 ± 0.5 μm), only separated by the plasma membranes.

### Transient averaging in 2D image stacks

Mean signal intensity for each ROI was calculated for each image yielding transients of Ca-dependent fluorescence (CaT) with data points every 18 ms (sampling interval, 55 frames per second). As even ROIs selected between cardiac myocytes were not free of Ca-dependent fluorescence changes that would allow recording of background fluorescence, Ca transients were recorded without background correction. During steady state stimulation, a period containing about 30 sequential Ca^2+^ transients was segmented by the stimulation cycle length (BCL, e.g., 250 ms). This resulted in fixed length time segments with data points occurring at varying positions within the individual segments. An averaged Ca transient was compiled from the data points by registering them on their relative position in the averaged cycle (see **Figure 2**). Based on a final temporal resolution of 2 ms and depending on the cycle length, 4–6 data points of different transients were averaged for each time point within the averaged cycle.

### Validation of the averaging technique

To validate the averaging technique we used single cardiac myocytes. Briefly, mouse hearts were perfused with a liberase-based Langendorff perfusion protocol as described previously (Ljubojevic et al., 2011) and cells were loaded with Fluo-8 (MoBiTec GmbH, Göttingen, Germany; 3.75 μM; 30 min) and placed on the stage of an inverted microscope equipped with a 40×/1.3 N.A. oil-immersion objective and a Zeiss LSM 700 confocal laser point scanning system (Zeiss, Jena, Germany). Excitation and emission wavelengths were 488 nm and >515 nm, respectively. Cells were field-stimulated via two platinum electrodes at 4 Hz.

We recorded CaT from single cells with an average sampling rate of 1.54 ms with a Zeiss LSM 510 in line scan mode. To simulate a transient with lower temporal resolution, we picked every 12th data point to achieve a sampling rate of 18.18 ms. Series of downsampled transients were then processed with our averaging technique to obtain an averaged transient with high temporal resolution. The restituted transient was compared to a high-resolution transient of the original trace. This yielded in a sampling interval of 1 ms with interpolated points and a median of 4 samples peer averaged data point.

### Data analysis

The images and averaged transients were analyzed with algorithms programmed in IDL (ITT) and Excel Visual Basic (Microsoft). We performed an automated transient analysis that delivered the peak amplitude and time to peak. The time constant of Ca decay (τ) was calculated with a mono-exponential fit; the starting point of the fitting curve was set to be 90% of the peak. All values were normalized to the index cell. In addition to the mean values for each local ROI, we calculated the mean of the absolute difference between two local ROIs as a measure of local variation. Large values thus indicate a high degree of variation in the respective parameter between the two ROIs. Local ROIs were compared along the longitudinal and the transversal axis within and between adjacent cardiac myocytes (see Results and **Figure 3**).

Statistical analysis of the data was performed using Graph Pad Prism. One-Way or Two-Way ANOVA with multiple *post-hoc* testing (Bonferroni) were performed where appropriate and values <0.05 were considered statistically significant.

## Results

### Intracellular dyssynchrony of cytosolic Ca removal in intact hearts

**Figure 1:**
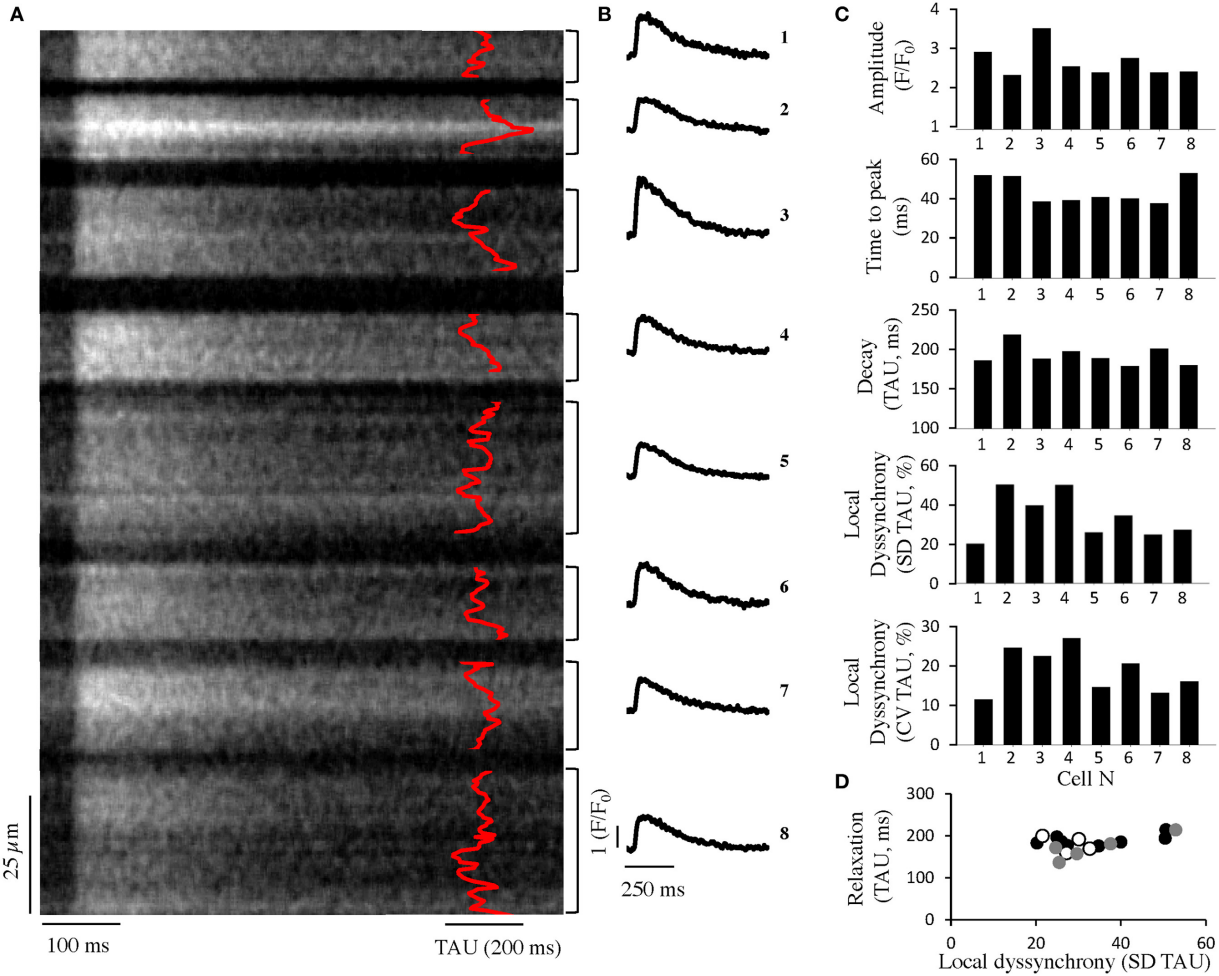
**Intracellular dyssynchrony of cytosolic Ca removal in intact hearts. (A)** Averaged confocal line-scan image of seven consecutive CaT during spontaneous activation (~1.4 Hz) from mouse ventricle in eight adjacent cells. The time constant of local Ca decay is superimposed in red. **(B)** Global Ca transients of the cells shown in **(A)**. **(C)** Quantification of the CaT amplitude (*top*), time to peak (*center, top*), relaxation (*center, bottom*) and local dyssynchrony (given as standard deviation (SD) of tau along the line as well as the coefficient of variation (CV), i.e., SD normalized to the global tau of the cardiomyocyte, *bottom*) of adjacent cells as obtained from a confocal line-scan (cells as depicted in **A**). **(D)** Quantification of relaxation and local dyssynchrony in adjacent cells from three different preparations (*white, black, gray*).

Using the approach previously applied in isolated cardiac myocytes (Hohendanner et al., 2013), we analyzed subcellular CaT in intact spontaneously beating Langendorff-perfused hearts using confocal line scan imaging of the epicardial layer (Figure 1). Similar to what we have previously described in isolated cardiomyocytes, intracellular cytosolic Ca removal was not synchronous in cardiomyocytes of the intact heart. Similar results were obtained at 3 Hz stimulation (data not shown). We also found variation in the amplitude, time to peak, and time constant of relaxation, tau, between adjacent cardiac myocytes. Whole cell (global) tau correlated with the degree of intracellular dyssynchrony in Ca removal in individual cells (Figure 1D; *p* < 0.05, *R* = 0.5 in 17 cells from 3 hearts).

### Variation of global cytosolic Ca transients between adjacent cardiac myocytes

**Figure 2:**
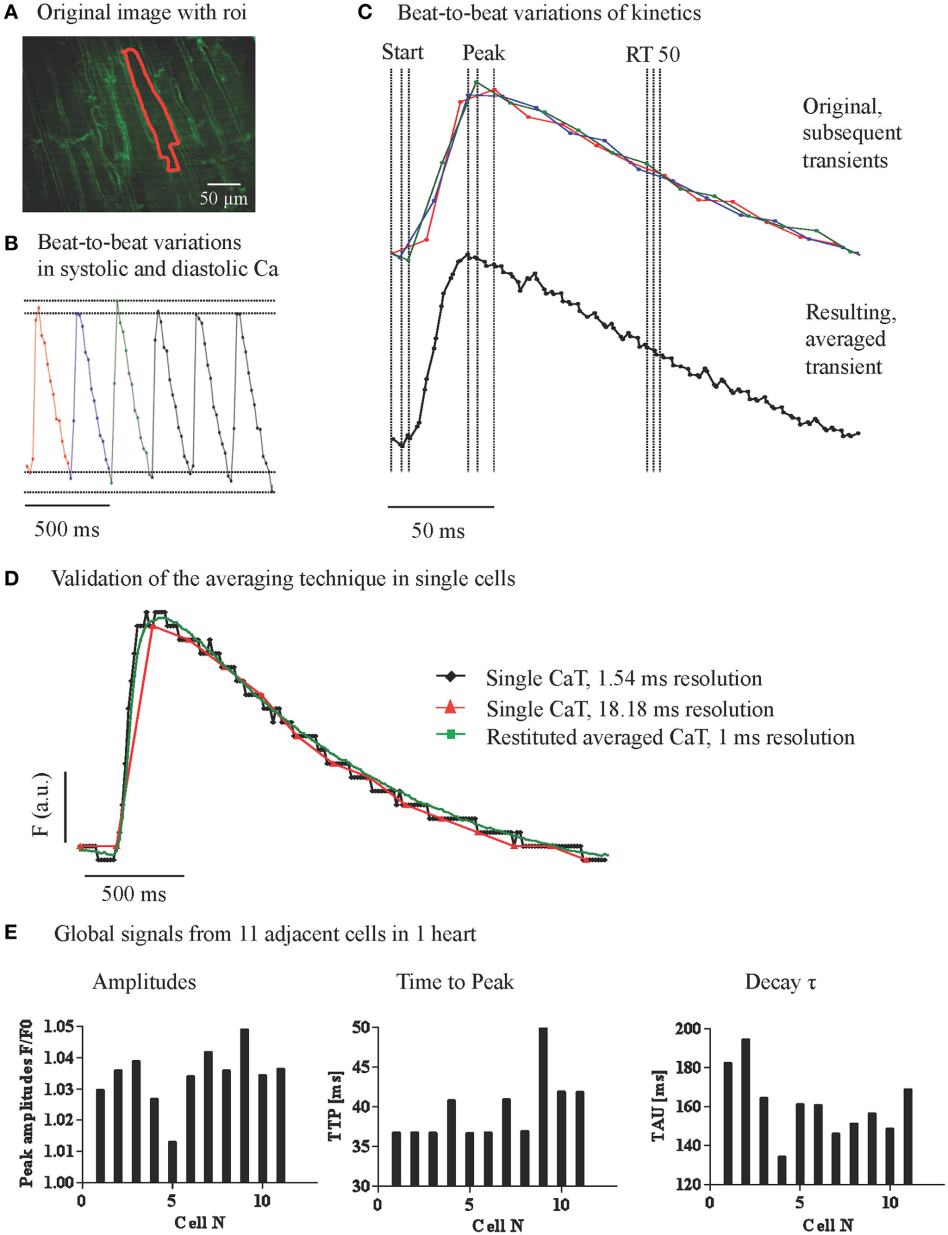
**Variations of global cytosolic Ca transients between adjacent cardiac myocytes. (A)** Confocal image of the epicardial layer of a Langendorff-perfused mouse hearts, loaded with Ca indicator Fluo-8 and the membrane dye Di-4-ANEPPS. Ca transients were recorded at 18 ms/frame with a Nipkow 2D confocal microscope and the Ca signals from adjacent myocytes were extracted. **(B)** The raw signal of Ca transients showed beat to beat fluctuations due to undersampling of the rapid change in intracellular Ca. **(C)** Data points from a series of sequential CaTs were registered on a matrix with 2.4 ms intervals to obtain an averaged transient with higher resolution. **(D)** A high-resolution line scan CaT was compared to a down sampled transient from the same data set (18.18 ms interval) and to the averaged transient resulting from the reaveraged down sampled trace. **(E)** Global signals from 11 adjacent cells within one preparation reconstructed with higher temporal accuracy revealed variations in CaT amplitudes, time to peak, and decay tau.

To quantify local CaT in adjacent cardiac myocytes with relation to their geometrical position, we used Nipkow disk 2D confocal microscopy. CaT extracted from the 2D image time series were limited by the temporal resolution of 18 ms, leading to beat to beat variation in the CaT amplitude and kinetics due to undersampling (Figure 2B) as well as blurring of the averaged signal due to different offsets of the data points in relation to the stimulus. With temporal registration of data points from sequential CaT within the cardiac cycle we obtained an averaged CaT with a temporal resolution of 2 ms (Figure 2C). On average 103 points per averaged transient with a mean interval of 2.42 ms (mean SD 1.34 ms) were taken for further analysis. To verify the validity of our approach we recorded Ca transients (line scans) in single cells with high temporal resolution to be able to compare the transients with a downsampled version of the same recording to simulate a recording from the whole heart with lower temporal resolution. We analyzed the down sampled transients with our averaging technique and found a high accuracy when compared with the original high-resolution transients (Figure 2D).

As shown in Figure 2E, and similar to our results from line scan imaging (Figure 1), amplitudes (not background corrected) as well as the kinetics of the whole cell CaT at steady state showed considerable variation between simultaneously recorded transients from adjacent cells amounting on average to 15–25% of the amplitude and 30–60% of the decay constant of the index cell.

### Variation of local cytosolic Ca transients related to the orientation in the myocardium

**Figure 3:**
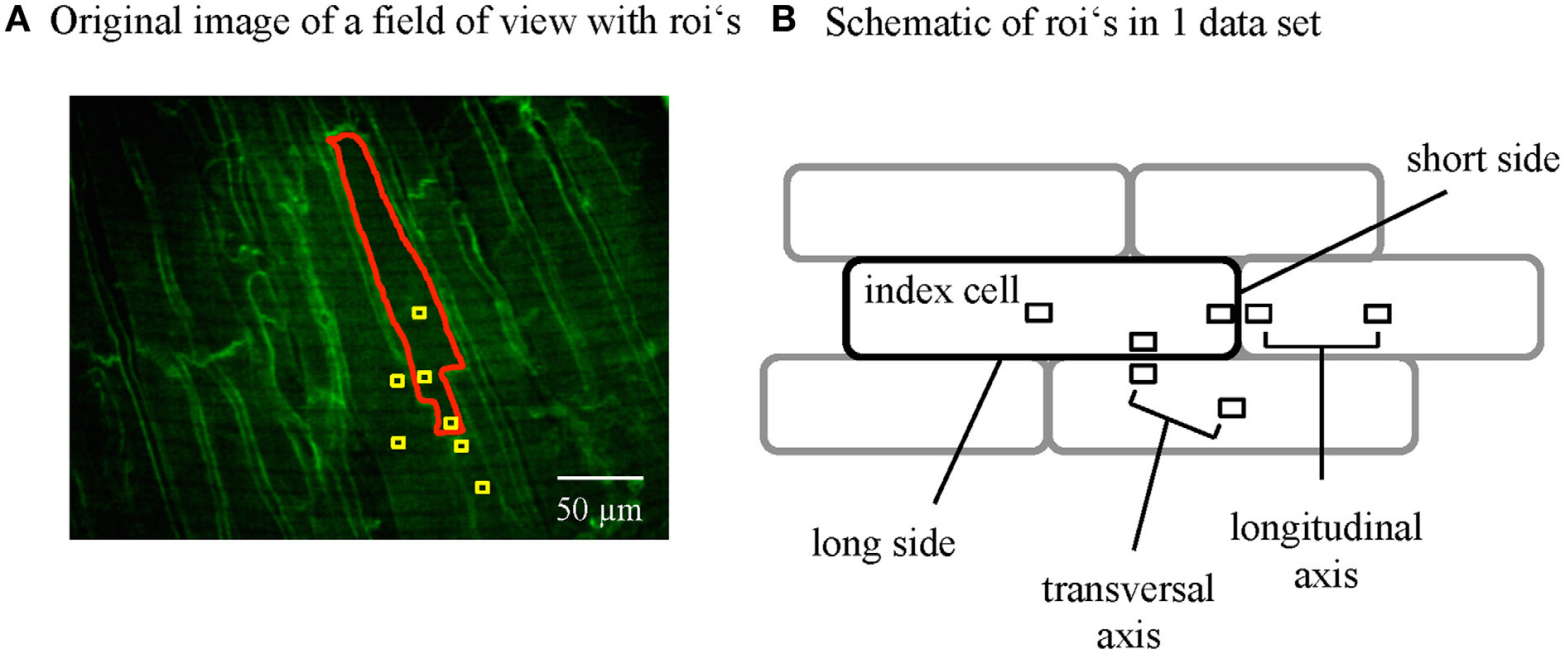
**Data analysis of subcellular Ca signals from adjacent myocytes. (A)** Regions of interest (ROIs) were positioned within the single cells where the Di-4-ANEPPS labeled the plasma membranes to visualize the cell borders. ROI's were positioned to extract the signal from the whole index cell and regions within the cells and the contact zones of adjacent cells at the short and the long axis following a standardized scheme **(B)**.

Based on our findings in whole cell (global) CaT from neighboring cells, we compared the variation in amplitude and kinetics of the CaT between two ROIs along the longitudinal axis and the transversal axis of the cardiac myocytes. We chose ROIs (central and subsarcolemmal) within an index cell and (subsarcolemmal) ROIs of neighboring cardiac myocytes to compare INTER- and INTRA-cellular variation along these orientations (Figure 3). The mean absolute difference in amplitude or kinetics between two ROIs was normalized to the global CaT of the index cell.

**Figure 4:**
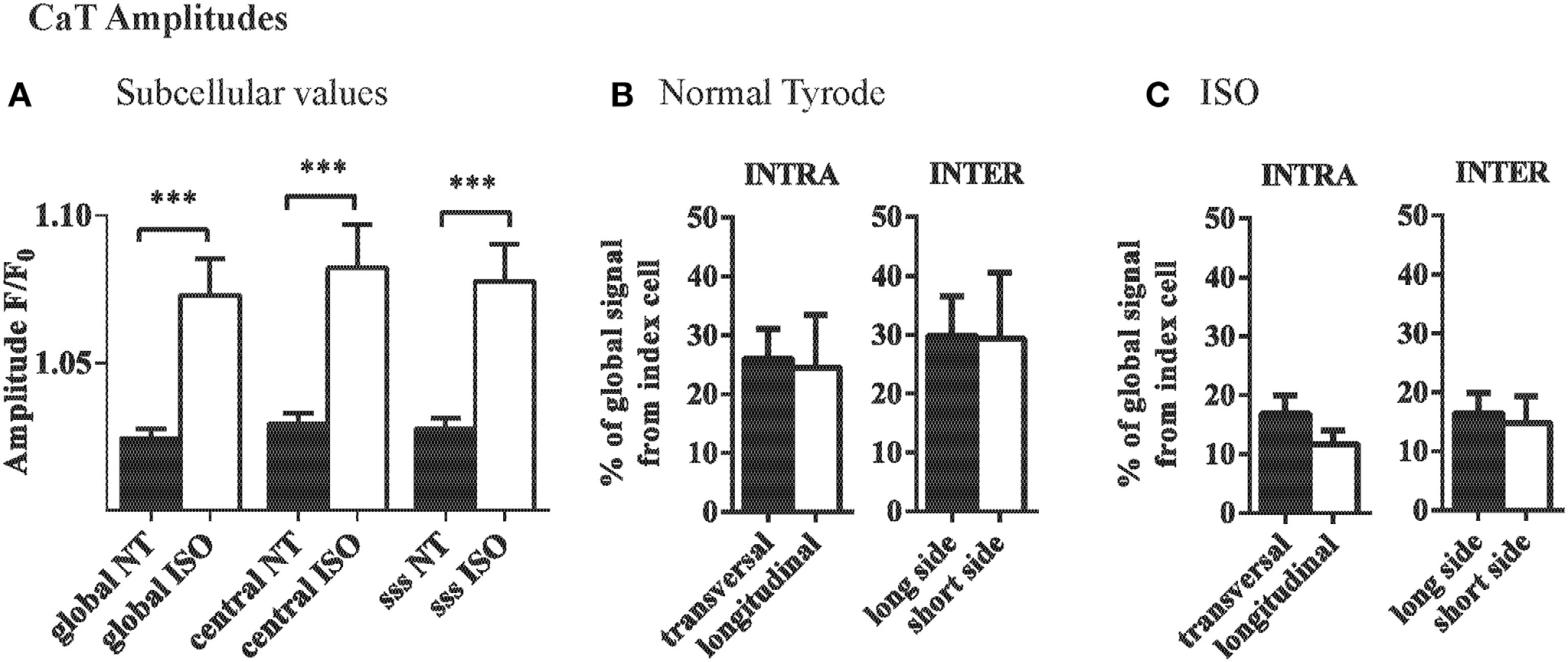
**INTRA- and INTERcellular variations in CaT amplitudes in the intact heart. (A)** Average CaT amplitudes were equal in different subcellular locations of myocytes and compared to the global signal of that cell. ISO increased the CaT amplitudes but did not change that pattern. **(B)** Variation in CaT amplitude between ROIs as quantified by the mean absolute difference was significantly higher in the longitudinal axis within the myocyte. Intercellular variation in amplitude was similar across the long and short side of the cardiac myocyte and compared to intracellular variation along the short axis. **(C)** The same overall pattern was found with ISO, where generally the differences between adjacent cells and within cells were smaller. ^***^p ≤ 0.001.

The mean amplitudes of the subcellular and central CaTs were not different in the index cell and were similarly increased by isoproterenol (Figure 4A). However, the variation of local CaT within the index cell (INTRA) along the longitudinal axis (between center and subsarcolemmal space at the short side) was 19.6 ± 4.9%, whereas no gradients (<0.01%) were detectable between the ROIs along the transversal axis within the cardiac myocytes (Figure 4B, left). Variation in CaT amplitude between the ROIs separated by the sarcolemma of adjacent cardiac myocytes (INTER) tended to be larger at the long vs. the short side (Figure 4B, right).

β-adrenergic stimulation with ISO decreased the variation of CaT amplitude in the longitudinal axis within the index cell (19.6 ± 4.8 F/F_0_ vs. 11.7 ± 2.4 F/F_0_ with ISO, ns). In the presence of ISO, the differences between the intercellular ROIs were equal between the longitudinal and the transversal axis (17.2 ± 4.8% and 16.4 ± 3.4%, respectively; Figure 4C).

**Figure 5:**
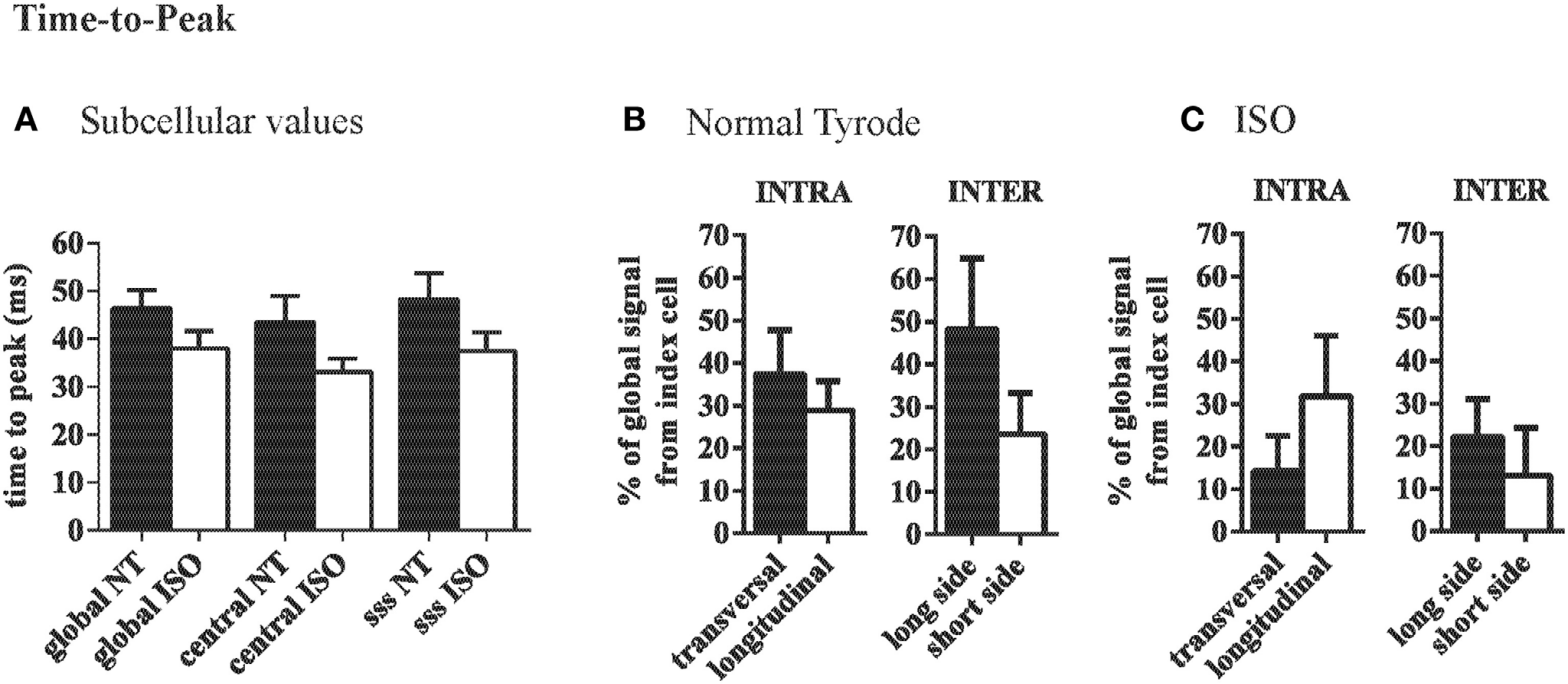
**Variation of time to peak Ca is similar across the long and short axis. (A)** Average time-to-peak values are comparable throughout a cell and are not significantly changed by ISO. **(B)** In NT variation of TTP is similar along the transversal and the longitudinal axis. Between adjacent cells there was a trend toward larger differences between adjacent cells at the short side compared to neighbors at the long side. This effect was eliminated by ISO **(C)**.

Within the index cells, the TTP of the CaT was not different between ROIs and did not significantly change with ISO (Figure 5A). Variation in TTP did not differ between the two ROIs along the longitudinal axis vs. the transversal axis of the index cell (Figure 5B). Variation in TTP across the membranes at the long side of adjacent cardiac myocytes tended to be higher (30.9 ± 9.8%) as compared to the differences between cells connected at their short side (10.9 ± 7.1%, Figure 5B). With β-adrenergic stimulation, this trend was eliminated (Figure 5C).

**Figure 6:**
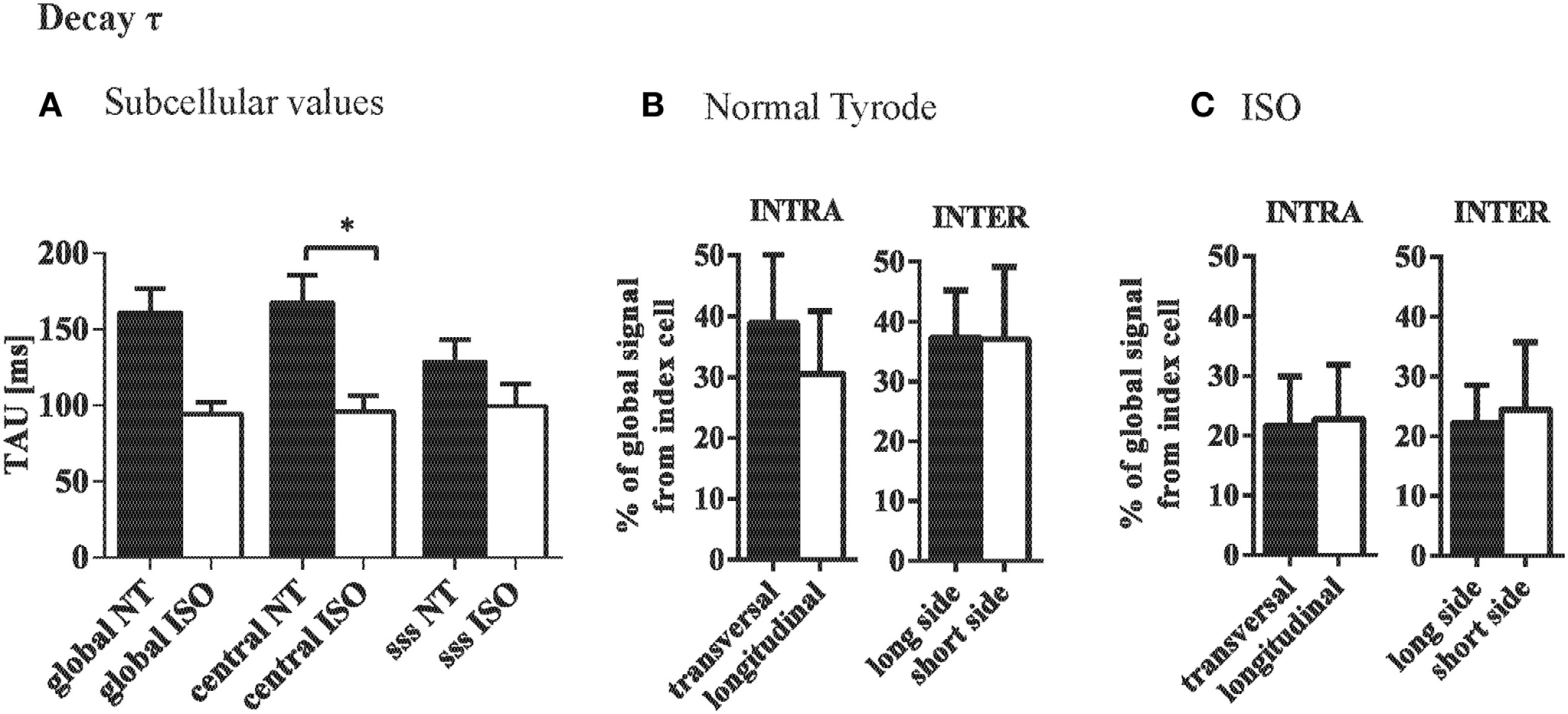
**Ca removal is synchronous at steady state stimulation. (A)** Decay of the CaT (tau) was not systematically different between central and subsarcolemmal (SSS) regions within cardiac myocytes. **(B)** Variation in tau was not depending on the orientation of the ROIs within or between neighboring cells. **(C)** ISO tended to increase the variation in tau but did not change the pattern found in NT. ^*^p ≤ 0.05.

The mean tau of the CaT at the central and subsarcolemmal region were comparable and significantly accelerated by ISO (Figure 6A). Variation in tau between the ROIs was independent of their orientation along the longitudinal axis or the transversal axis within or between adjacent cells (Figure 6B), and similar was observed in the presence of ISO (Figure 6C).

## Discussion

In this study we show that in the intact, regularly beating healthy heart there is considerable variation in the amplitude and kinetics of simultaneously recorded Ca transients of adjacent cardiac myocytes. While the orientation of the cardiac myocytes toward each other (transversal vs. longitudinal) had no influence on the intercellular variation, the degree of dyssynchronous Ca removal in individual cells *in vivo* contributes to the variation in Ca decay between cells (Figure 1). We present a new approach to record and analyze Ca data from intact mouse hearts from 2D time series resulting in averaged Ca transients with high spatial and temporal resolution. Intra- and intercellular variation in Ca^2+^ transient amplitude and kinetics during steady state stimulation as presented in this study describes a new modality in EC coupling.

The function of the heart as a pump is the result of an orchestrated sequence of activation and relaxation of the muscle fibers that constitute the myocardium. The rapid spread of electrical activation facilitated by the specialized conduction system and intercellular gap junctions between cardiac myocytes has been recognized as an important regulator of the heart's efficacy as a pump, and electrical resynchronization therapy has evolved as an important therapeutic approach in selected patients (Kirk and Kass, 2013). However, electrical activation is only the first step in ECC, initiating the AP. Previous clinical studies suggested that systolic and diastolic mechanical dyssynchrony may exist despite overall rapid electrical activation (Yu et al., 2006, 2007). At the level of the cardiac myocyte, intracellular Ca regulation is the main modulator of function as it determines contractile force by myofilament activation but also electrical excitability by shaping the AP. The intracellular Ca transient is the summation of localized Ca release events triggered by Ca influx through voltage gated Ca channels (Ca-induced Ca-release, CICR). We and others have previously shown that the synchrony of intracellular Ca release events during the regular cardiac cycle is a determinant of the efficacy of ECC in cardiac remodeling (Louch et al., 2004; Song et al., 2006; Heinzel et al., 2008). Ca removal from the cytosol is also regulated locally, and dyssynchrony in Ca removal is associated with slowed Ca decay and relaxation in isolated cardiac myocytes (Hohendanner et al., 2013).

At the whole heart level, synchrony of Ca transients has to be considered within but also between adjacent cardiomyocytes. Previous study provided evidence that the level of synchrony of spontaneous diastolic Ca release between cells is of relevance for arrhythmogeneity in the intact heart (Brunello et al., 2013), but variation in electrically stimulated Ca transients at steady state has not been described. In the present study, we demonstrate that different degrees of subcellular dyssynchrony in cytosolic Ca removal of individual cells are also present in the intact heart. Furthermore, the degree of intracellular dyssynchrony contributes to the variation in the kinetics of global Ca decay between adjacent cardiac myocytes (Figure 1D). Our results corroborate the notion of a role for subcellular dyssynchrony in shaping global cardiac myocyte Ca decay. At the same time the data suggest that dyssynchrony in Ca decay between cardiac myocytes in the regular beating heart is not negligible and thus may be a modulator of contractile efficacy. Larger discrepancies in Ca kinetics and resulting mechanical activity may lead to local strain patterns between loaded and unloaded cardiac myocytes as has been suggested to occur on a smaller myofilament-scale level (Edman, 1980). In addition, resulting mechanical heterogeneity may be arrhythmogenic through mechano-electrical feedback as previously modeled for larger ischemic regions during acute ischemia (Jie et al., 2010). It therefore seems reasonable to assume mechanisms that stabilize synchronous Ca handling and activity in adjacent cardiac myocytes.

The Ca transient is to some degree shaped by the AP via its influence on transsarcolemmal Ca currents (L-type Ca currents, sodium calcium exchanger, and plasmalemmal Ca pumps). APs of adjacent cells are coupled via gap junctions formed between cardiac myocytes which can pass ionic currents in a highly regulated way (Kurtenbach et al., 2014). At the short side of the cell we expect to have the vast majority of gap junctions formed between the cells, contributing to the physiological anisotropy of electrical propagation by rapid activation of APs along the longitudinal axis of the cardiac myocytes (Severs et al., 2008; Veeraraghavan et al., 2014). It has been suggested that Ca exchange between adjacent cardiac myocytes can occur during Ca wave propagation (Takamatsu et al., 1991; Li et al., 2012). While the probability of Ca wave transmission between adjacent cells in the intact heart was low at baseline it was modifiable by isoproterenol (increase) and the gap junction uncoupler heptanol (Lamont et al., 1998) and increased in conditions of Ca overload (Kaneko et al., 2000). Recently it has been suggested that Ca wave propagation is much more likely to occur in cardiac myocyte pairs connected side to side (Li et al., 2012), indicating that Ca exchange may depend on the geometric arrangement of the cells. However, Ca waves do not occur in healthy cardiac myocytes, and the transsarcolemmal association of Ca signals during steady state conditions has not been studied in the intact heart.

To quantify directional local Ca gradients in adjacent cardiac myocytes in the intact heart, in the present study we have developed a new approach to register 2D high resolution confocal images from sequential Ca transients recorded at the optimum speed of our system of 18 ms/frame to a matrix of an averaged Ca transient with a much higher temporal resolution. This allowed a more accurate quantification of local Ca transient kinetics in the averaged transient. Intrinsic to the method, it is not sensitive to sporadic Ca events (sparks) or irregular beat-to-beat changes, as sporadic events average out with registration of multiple images at the same time point in the averaged transient.

Quantification of the variation of local Ca transient between two ROIs within the same cell revealed comparable variation of about 25% in the local Ca transient amplitudes in the longitudinal and transverse direction. Enhanced Ca release from the sarcoplasmic reticulum in the presence of isoproterenol did not significantly affect relative Ca transient amplitude variation. It has been shown previously that the diffusion distance between RyRs along the long axis is shorter than in the transversal direction (Chen-Izu and William Balke, 2006; Izu et al., 2006), which also explains why the spread of Ca waves is facilitated along the long axis (Li et al., 2012). However, in the present study, during steady state stimulation, we observed that time to peak Ca within cardiac myocytes was not different in longitudinal and transversal direction, likely reflecting the dense three-dimensional t-tubule network in rodents, which allows synchronous activation of CICR in the healthy heart (Cannell and Soeller, 1998; Heinzel et al., 2002). Ca decay (tau) on average varied by 20% (Figure 5B) independent of the orientation along the myocyte's axis and comparing well with the 19% average variation of local tau found in longitudinal line scan recordings in healthy isolated mouse cardiac myocytes (Hohendanner et al., 2014).

We compared the variation of local Ca transients separated only by the plasma membranes of adjacent cardiac myocytes (INTER) at the long and short sides. Variation in Ca amplitude was considerable within the narrowly placed ROIs in adjacent cardiac myocytes (Figure 4B). Ca transient amplitude measurements in our study were not corrected for background (see Methods), but variation of the Ca amplitude between adjacent cardiac myocytes was comparable to the Ca gradients within individual cardiac myocytes (Figure 4B), thus a confounding effect related to differences in background between adjacent cardiac myocytes is less likely.

In our conditions in healthy mouse hearts, the variation in amplitude and kinetics between adjacent cardiac myocytes (INTER) did not significantly differ from the variation within individual cells (INTRA), and was similarly affected by isoproterenol. Therefore, at basal conditions, the shape of the Ca transients seems to be mainly determined by the Ca regulation properties of the individual cardiac myocytes with no additional contribution of cell-to-cell coupling to the heterogeneity of local Ca transients in the intact heart.

Several mechanisms may determine the degree of synchrony in cytosolic Ca removal within and thus between cardiac myocytes: Transport of Ca from the cytosol to the nucleus is the main mechanism of cytosolic Ca removal in cardiac myocytes. Recently, the co-expression of SERCA isoforms with different Ca transportation properties in cardiomyocytes has been described (19962989). It is possible that a distinct pattern of SERCA isoform expression or their local post-translational regulation contributes to local dyssynchronous Ca removal. Furthermore, we have recently shown that regions with increased mitochondrial density show an increased rate of cytosolic Ca removal. In this context, at least in rodents, the distribution of the sarcolemmal N/Ca exchanger does not contribute to dyssynchronous Ca removal (Hohendanner et al.). Finally local cytosolic Ca decay may be influenced alterations in local Ca buffering or diffusion constraints. All of these factors are influenced by cardiac remodeling and may contribute to dyssynchrony.

We have performed all experiments at RT to reduce dye leakage out of the cells and improve longevity of the hearts (Wasserstrom et al., 2010) which caused us to adjust the stimulation frequencies used in the present study. It has been shown that the propensity toward arrhythmogenic events in the heart is shifted toward shorter BCL's with reduced temperature caused by increased CaT duration (Aistrup et al., 2006). However, it also has been shown that in whole heart preparations Ca cycling behavior at RT is qualitatively highly comparable to Ca cycling at physiological temperature (Wasserstrom et al., 2010). A shift in temperature will shift the relative contribution of NCX toward relaxation at the expense of SERCA at different degrees in different species (Mackiewicz and Lewartowski, 2006), but we expect this to be small in mice with SERCA being the major contributor to relaxation. In addition, in the present study, fluorescence signals were not background corrected due to technical limitations outlined in Methods. However, differences in background fluorescence do not explain the observed variance in Ca transient relaxation. In Figure 1 we have calculated the variance in Ca reuptake as standard deviation (SD) as well as coefficient of variation (CV) related to the mean tau of the cardiomyocyte. While CV as a relative measure shows a similar degree of differences between adjacent cells as SD, calculating CV assumes a linear interdependence of SD and mean tau within a cardiomyocyte which remains hypothetical.

## Summary and perspective

We present a novel approach to quantify 2D spatial heterogeneities in Ca transients with high temporal accuracy in the intact Langendorff perfused murine heart. In regularly beating hearts we observed considerable variation in the simultaneously recorded Ca transients of adjacent cardiac myocytes. Variation in Ca decay was related to the intracellular dyssynchrony in Ca reuptake. In near physiological conditions, the degree of dyssynchrony did not seem to be related to the orientation of adjacent cardiac myocytes toward each other, but rather to intrinsic factors of the cardiac myocytes. In pathological conditions, such as acute ischemia or cardiac remodeling, intracellular Ca regulation is disturbed and responds to alterations in the micro-environment of the cell. Heterogeneity of Ca transients in adjacent cardiac myocytes at steady state requires further study as it may represent a new modality influencing syncytial myocardial function.

### Conflict of interest statement

The authors declare that the research was conducted in the absence of any commercial or financial relationships that could be construed as a potential conflict of interest.
